# Regulation of Eosinophil Recruitment and Activation by Galectins in Allergic Asthma

**DOI:** 10.3389/fmed.2017.00068

**Published:** 2017-05-31

**Authors:** Savita P. Rao, Xiao Na Ge, P. Sriramarao

**Affiliations:** ^1^Department of Veterinary and Biomedical Sciences, University of Minnesota, St. Paul, MN, United States

**Keywords:** eosinophils, cell trafficking, galectins, airway recruitment, allergic airway inflammation, asthma

## Abstract

Eosinophils are differentiated granulocytes that are recruited from the bone marrow to sites of inflammation *via* the vascular system. Allergic asthma is characterized by the presence of large numbers of eosinophils in the lungs and airways. Due to their capacity to rapidly release inflammatory mediators such as cytokines, chemokines, growth factors, and cytotoxic granule proteins upon stimulation, eosinophils play a critical role in pro-inflammatory processes in allergen-exposed lungs. Identifying key players and understanding the molecular mechanisms directing eosinophil trafficking and recruitment to inflamed airways is a key to developing therapeutic strategies to limit their influx. Recent studies have brought to light the important role of glycans and glycan binding proteins in regulating recruitment of eosinophils. In addition to the role of previously identified eosinophil- and endothelial-expressed adhesion molecules in mediating eosinophil trafficking and recruitment to the inflamed airways, studies have also indicated a role for galectins (galectin-3) in this process. Galectins are mammalian lectins expressed by various cell types including eosinophils. Intracellularly, they can regulate biological processes such as cell motility. Extracellularly, galectins interact with β-galactosides in cell surface-expressed glycans to regulate cellular responses like production of inflammatory mediators, cell adhesion, migration, and apoptosis. Eosinophils express galectins intracellularly or on the cell surface where they interact with cell surface glycoconjugate receptors. Depending on the type (galectin-1, -3, etc.) and location (extracellular or intracellular, endogenous or exogenously delivered), galectins differentially regulate eosinophil recruitment, activation, and apoptosis and thus exert a pro- or anti-inflammatory outcome. Here, we have reviewed information pertaining to galectins (galectin-1, -3 -9, and -10) that are expressed by eosinophils themselves and/or other cells that play a role in eosinophil recruitment and function in the context of allergic asthma and their potential use as disease biomarkers or therapeutic targets for immunomodulation.

## Introduction

Eosinophils are the predominant granulocytic leukocytes present in allergic airways, and eosinophilia is the hallmark of airway inflammation in asthma (1–3). Although constituting only a small fraction of circulating white blood cells under healthy conditions, patients with allergic airway inflammation and asthma exhibit significantly higher numbers of eosinophils in peripheral blood (4). Mature eosinophils are formed from progenitor stem cells in the bone marrow and released into circulation under the tight regulation of interleukin (IL)-5 (5). During disease conditions such as allergic asthma, they undergo proliferation and priming in response to specific stimuli (6). Indeed, peripheral blood eosinophils from allergic asthmatics have been shown to demonstrate spontaneously enhanced production of reactive oxygen species, increased chemotaxis, and diminished apoptosis relative to eosinophils from normal subjects (7). Once recruited to sites of inflammation, eosinophils exert their pathological and immunomodulatory effects. Eosinophils contain cationic granule proteins that have been shown to exert highly cytotoxic effects such as production of reactive oxygen species, desquamation, and lysis of airway epithelial cells, as well as synthesis of remodeling factors by epithelial cells [reviewed in Ref. (8)]. In addition, eosinophils are a source of various cytokines, chemokines, and growth factors that are either preformed or synthesized and secreted upon activation (9). Thus, identifying key players involved in selective eosinophil recruitment and understanding their role in supporting this event is critical for the identification of therapeutic targets.

**Figure 1:**
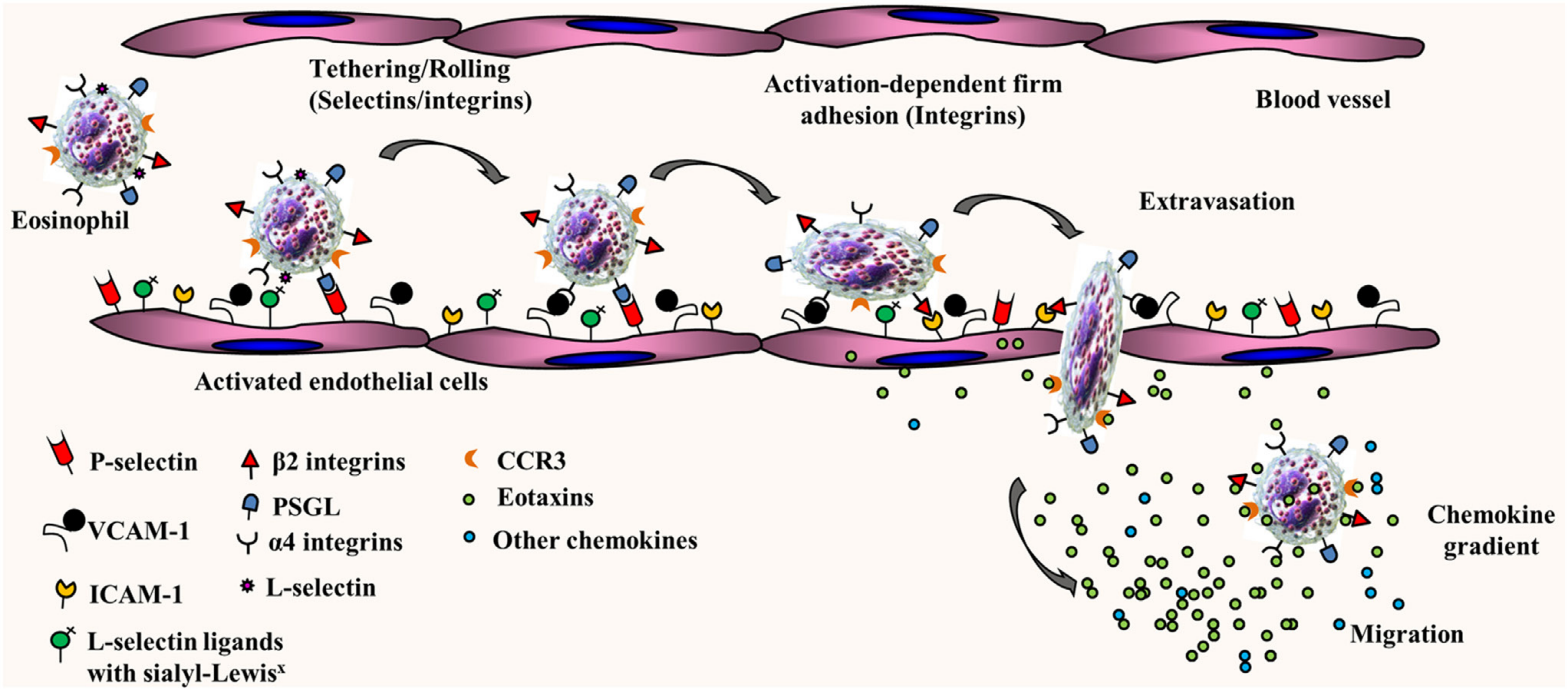
Multistep paradigm of eosinophil trafficking under conditions of flow. Eosinophil rolling along the inflamed and activated vascular endothelium is supported by eosinophil-expressed L-selectin, α4β1, and P-selectin glycoprotein ligand-1 (PSGL-1) and endothelial-expressed vascular cell adhesion molecule (VCAM)-1 and P-selectin. Activation-dependent stable adhesion of eosinophils to the vascular endothelium is mediated by α4β1/VCAM-1 and αMβ2/intercellular adhesion molecule (ICAM)-1 interactions. Extravasation or trans-endothelial migration is under the control of chemokines (eotaxins and other chemokines).

The trafficking of primed mature eosinophils from the blood stream into inflamed tissues such as the lung is finely regulated by adhesion molecules, several cytokines, and chemokines with overlapping functions. We and others have demonstrated that eosinophil trafficking under conditions of flow involves a multistep paradigm, which includes initial tethering/rolling followed by activation-dependent firm adhesion and chemoattractant-induced transmigration into extravascular sites of inflammation [as reviewed in Ref. (10–12), see Figure 1]. Studies with mouse and/or human eosinophils have shown that rolling along the vascular endothelium is supported by L-selectin, very late antigen-4 (α4β1), and P-selectin glycoprotein ligand-1 (PSGL-1). On the endothelial side, in addition to the α4 ligand vascular cell adhesion molecule (VCAM)-1, eosinophil rolling under physiological conditions of flow is mediated by P-selectin, the ligand for PSGL-1 (13, 14). Apart from mediating inter-eosinophil interaction under physiological flow conditions (13), L-selectin has been shown to interact with endothelial ligands bearing sialyl-Lewis^x^ epitopes, such as CD34, to play a role in recruitment of eosinophils to allergic lungs in mice (15). Rolling of human eosinophils under conditions of physiologic blood flow in post-capillary venules does not appear to be dependent on E-selectin, albeit these cells can roll on immobilized E-selectin under sub-shear flow rates (16). While an overview of eosinophil trafficking is depicted in Figure 1, it must be noted that human and mouse eosinophils are likely to utilize different vascular adhesion molecules during cell trafficking due to species-specific differences in expression of these molecules in response to inflammatory stimuli. Activation-dependent stable adhesion of eosinophils to the vascular endothelium is mediated by α4β1/VCAM-1 and integrin αMβ2/intercellular adhesion molecule (ICAM)-1 interaction. Trans-endothelial migration is under the control of pro-inflammatory chemokines, predominantly eotaxins, and the eotaxin receptor, C–C chemokine receptor type 3 (CCR3). In addition to these key players, there are several other molecular events that support the dynamic recruitment of eosinophils into extravascular sites such as chemoattraction by molecules other than eotaxins [e.g., PAF, C–C motif chemokine ligand 5 (CCL5), C–C motif chemokine ligand 3 (17), C3a and C5a (18), and serotonin/5-hydroxytryptamine (19)] as well as release and activation of matrix metalloprotease-9, which enables eosinophil migration through the extracellular matrix (20–22). Of particular interest in our laboratory is the role played by cell surface-expressed complex carbohydrate structures such as heparan sulfate proteoglycans (23–25) as well as branched *N*-glycans (26) and their galectin ligands (27–30) in leukocyte/eosinophil–endothelial interactions during cell trafficking and migration/recruitment.

Galectins are a family of animal lectins that specifically bind to β-galactosides in cell surface glycoproteins and glycolipids or in free form (31, 32). Fifteen galectins have been identified in mammals to date and are classified into three groups based on their structure. The three groups are (i) the prototypic galectins (Gal-1, -2, -5, -7, -10, -11, -13, -14, and -15), which contain one carbohydrate recognition domain (CRD), (ii) the tandem-repeat galectins (Gal-4, -6, -8, -9, and -12), which contain two homologous CRDs within a single polypeptide, and (iii) chimeric galectins (Gal-3), which have a single CRD and a large amino-terminal domain. While many galectins exhibit wide tissue distribution, some are tissue specific such as Gal-7, which is predominantly expressed in stratified epithelium, and Gal-4, which is mostly expressed in mouse gastrointestinal tissue (33, 34). Galectins are largely present in the cytoplasm and nucleus within cells. However, despite the lack of a classical signal sequence for export, several galectins are also present extracellularly either interacting with cell surface glycans or in a soluble form (e.g., in bronchoalveolar lavage fluid and serum) (31). Glycoproteins on the cell surface (including adhesion receptors and cytokine receptors) containing branched *N*-glycans with *N*-acetyllactosamine residues function as epitopes for galectin binding, especially for Gal-1 and Gal-3 (35). Galectins form complexes (dimers or oligomers) that crosslink cell surface glycoprotein receptors to form dynamic lattices that can regulate responses and properties of these receptors (32). Depending on the type (e.g., Gal-1 or Gal-3), location and binding partners (intracellular/non-carbohydrate or extracellular/cell surface glycans), and concentration, galectins positively or negatively regulate various cellular events such as signal transduction, cell differentiation and maturation, production of cytokines and growth factors, trafficking and recruitment, and apoptosis during acute and chronic inflammation [as reviewed in Ref. (36, 37)]. Thus, galectins play a role in various aspects of health and disease such as normal development, innate immunity, inflammation, cardiovascular disease, obesity, type 2 diabetes, and cancer [as reviewed in Ref. (31, 34, 36, 38–40)]. This review is focused on galectins that are expressed by eosinophils themselves and/or other cells that play a role in eosinophil recruitment and function, i.e., Gal-1, -3, -9, and -10 in the context of allergic asthma.

### Galectin-3

Gal-3 [same as IgE-binding protein (εBP), CBP35, or Mac-2] was first identified in rat basophilic leukemia cells (41). This lectin is a chimeric galectin containing a single CRD connected to a non-lectin domain rich in proline, glycine, and tyrosine residues with the ability to self-aggregate and thus function bivalently or multivalently (42). Gal-3 is expressed by most tissues, including the lung, all types of epithelia, and most inflammatory cells (mast cells, neutrophils, monocytes/macrophages, eosinophils, and T cells) (43). It is extensively characterized and has been shown to exert diverse functions depending on its location (extracellular or intracellular) that suggest a positive regulatory role for this lectin during inflammation. Extracellular Gal-3 affects cellular events ranging from cell activation [mast cells (44), neutrophils (45), macrophages (46), and lymphocytes (47)] to adhesion [neutrophils (48)], migration [macrophages (49)], and apoptosis [mast cells (50) and T cells (51)]. Intracellular Gal-3 has been shown to participate in pre-mRNA splicing (52), phagocytosis by macrophages (53), and exert antiapoptotic activity in T cells *via* interaction with Bcl-2 (54) as well as in peritoneal macrophages (55). Studies in Gal-3-deficient mice have provided strong evidence for the pro-inflammatory role of Gal-3 in various acute models of inflammation (55–58) including allergic disorders such as asthma (59) and atopic dermatitis (60).

**Figure 2:**
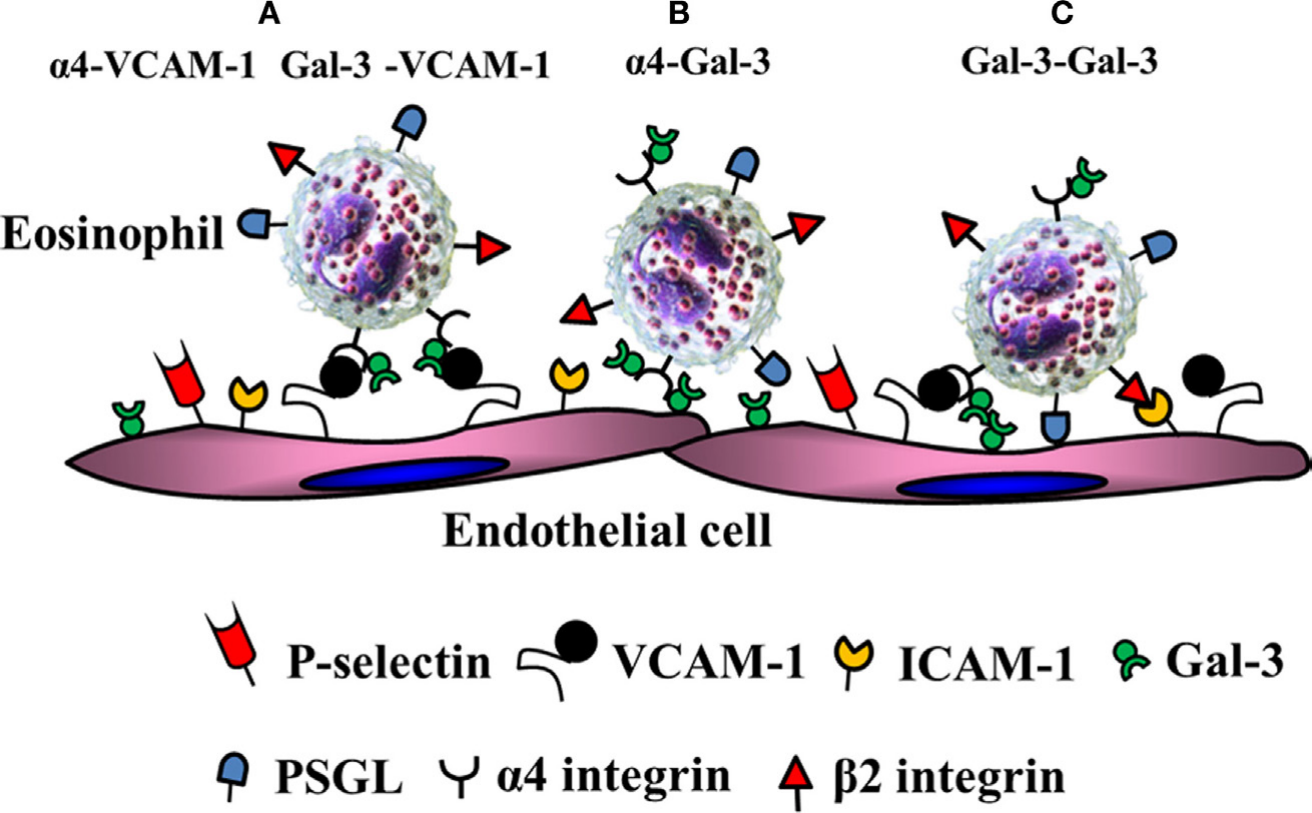
Gal-3-mediated eosinophil–endothelial interactions during cell trafficking. Gal-3 is present on the surface of eosinophils bound to α4. **(A)** In addition to α4β1/vascular cell adhesion molecule (VCAM)-1 interactions, eosinophil-expressed Gal-3 can independently interact with EC-expressed VCAM-1. **(B)** Eosinophil-expressed α4 can interact with EC-expressed Gal-3. **(C)** Eosinophil-expressed Gal-3 can bind to EC-expressed Gal-3.

Acute allergen exposure was shown to result in increased recruitment of Gal-3-expressing inflammatory cells (macrophages and eosinophils) to the airways and elevated levels of soluble Gal-3 in the bronchoalveolar lavage fluid of wild-type mice (59). On the other hand, allergen-challenged Gal-3-deficient mice exhibited significantly decreased airway eosinophil recruitment and an overall reduction in airway inflammation (decreased mucus secretion, airway hyperresponsiveness, and Th2 responses) relative to the wild-type mice. In support of this, studies from our laboratory showed that eosinophils from allergic subjects express elevated levels of Gal-3 on the cell surface and exhibit increased adhesive interactions (rolling and firm adhesion) on VCAM-1 compared to cells from normal subjects under conditions of flow in a Gal-3-dependent manner (27). Additionally, we showed that inflamed human endothelial cells express elevated levels of Gal-3 on the cell surface and that blockade of endothelial Gal-3 with specific antibodies inhibits eosinophil rolling and adhesion. At a molecular level, Gal-3 was found to interact with α4 integrin *via* its CRD and showed co-localized expression with α4 on the cell surface of eosinophils from allergic subjects. In addition, eosinophil-expressed Gal-3 interacted with endothelial Gal-3. Self-association to homodimerize or form multivalent complexes is a characteristic feature of Gal-3 (61). Since galectins do not contain a classical signal sequence or a transmembrane domain but are still present extracellularly, it is likely that eosinophil-derived Gal-3 is presented on the cell surface anchored to glycosylated residues on α4 *via* its CRD (based on the blockade of these interactions by lactose) after exiting the cell where it is then able to mediate eosinophil rolling and adhesion on VCAM-1 and Gal-3 as depicted in the schematic shown in Figure 2. Studies with total leukocytes from bone marrow of Gal-3-deficient mice further support these findings (28). While cells from wild-type mice demonstrated increased rolling on VCAM-1 and Gal-3 that was specifically inhibited by lactose, rolling of Gal-3-deficient cells on both these endothelial-expressed adhesion molecules was significantly lower and unaffected by lactose. Further, in a model of chronic asthma, there was significantly decreased eosinophil infiltration associated with an overall reduction in the development of a Th2 phenotype and diminished remodeling of the airways (reduced mucus secretion, subepithelial fibrosis, smooth muscle thickness, and peribronchial angiogenesis) in Gal-3-deficient mice compared to wild-type mice (28). In addition to integrin receptors, Gal-3 has been shown to bind to CD66b (CEACAM8), a single chain, highly glycosylated member of the Ig superfamily expressed exclusively on activated human eosinophils and induce cell adhesion, superoxide production and degranulation (62).

The identification of novel procedures to culture mature primary murine eosinophils from bone marrow *in vitro* (63) has enabled further understanding of the role played by eosinophil-expressed galectins in cell trafficking and activation. Eosinophils cultured from bone marrow of Gal-3-deficient mice showed significantly less rolling on VCAM-1 under conditions of flow, which was also associated with decreased adhesion on ICAM-1 likely due to the inherently decreased expression levels of αM integrin (29), which is necessary for firm adhesion of eosinophils. Interestingly, αM integrin functions as a binding partner for extracellular Gal-3 in macrophages (64). While only hypothetical, it is possible that expression of Gal-3-binding integrin receptors on the cell surface may be regulated by intracellular Gal-3. Adherent Gal-3-deficient cells demonstrate limited ability to spread and form membrane protrusions, which are important cytoskeletal changes that enable stable adhesion and directed movement of cells toward a chemokine gradient (65). Consistent with this observation, Gal-3-deficient eosinophils show reduced migration toward eotaxin-1 despite normal levels of CCR3 (the eotaxin receptor) expression relative to wild-type eosinophils. Studies have shown that intracellularly, Gal-3 in fibroblasts is phosphorylated and that phosphorylation is required for localization of Gal-3 at the cell periphery [an event that has been shown to be required for cell motility (57)] as well as for secretion of Gal-3 (66). Inflammatory mediators such as eotaxin-1 induce secretion of Gal-3 by wild-type murine eosinophils into the culture supernatant (29), which might be one of the mechanisms by which intracellular Gal-3 is presented on the cell surface to promote cell trafficking *via* interaction with endothelial ligands. It is possible that regulation of eosinophil trafficking and migration by intracellular Gal-3 as well as its secretion during conditions of inflammation may involve phosphorylation.

As indicated earlier, galectins bind to β-galactoside epitopes found in *N*- and *O*-glycan modifications of glycoproteins and glycolipids (31, 32). Thus, galectin binding to glycoproteins is determined by the number of glycosylation sites and activity of various glycosyltransferase enzymes of the Golgi complex [reviewed in Ref. (32)]. UDP-*N*-acetylglucosamine:α-6-d-mannoside β1,6 *N*-acetylglucosaminyltransferase V (Mgat5) generates intermediate *N*-glycans that are further extended by the addition of *N*-acetyllactosamine units resulting in high affinity ligands for Gal-3 (67). Studies using mice deficient in this enzyme further confirmed the involvement of Gal-3 and the requirement for finely regulated *N*-glycosylation of surface glycoproteins in eosinophil trafficking and recruitment (26). *In vivo* allergen-challenged Mgat5-deficient mice demonstrated significantly decreased recruitment of eosinophils to the airways along with reduced Th2 cytokines and airway mucus production compared to their wild-type counterparts. *In vitro* eosinophils from Mgat5-deficient mice showed decreased rolling and adhesion on Gal-3 and VCAM-1. It is well known that *N*-glycosylation regulates the biological functions of integrins (clustering, adhesion, and migration) and that changes in glycosylation can affect these functions [as reviewed in Ref. (68)]. Thus, in the absence of Mgat5, not only are α4β1-VCAM-1 interactions likely to be affected but also the ability of Gal-3 to bind to α4, resulting in decreased eosinophil recruitment. Although it is currently not known whether expression of glycosyltransferases involved in generating galectin-specific ligands are elevated during allergic asthma, there is evidence for regulation of expression of glycosyltransferases (α1,3/4-fucosyltransferase and α2,3-sialyltransferases genes) in the lung by pro-inflammatory cytokines (IL-6, IL-8, and TNF-α) in diseases such as cystic fibrosis, which contributes to disease pathogenesis by increasing the number of sialyl-Lewis^x^ epitopes and thus favoring attachment of *P. aeruginosa* (69).

In contrast to the pro-inflammatory role ascribed to endogenous Gal-3, studies by other investigators have shown that gene therapy with Gal-3-encoding plasmid DNA can suppress eosinophil infiltration and normalize pulmonary function in acute as well as chronic settings of allergic asthma (70, 71) by negatively regulating gene expression of suppressors of cytokine signaling 1 and 3, which play an important role in controlling the Th1–Th2 balance (72). Further, these studies suggest that administration of exogenous Gal-3 could potentially serve as a therapeutic tool for allergic asthma. Differences in the effects noted with exogenous Gal-3 delivery versus endogenous Gal-3 expression may be multifactorial such as the cells in which the protein is expressed endogenously during allergic asthma versus those targeted by the delivery of exogenous Gal-3, the concentration at which Gal-3 is present in the local milieu, as well as the activity and mode of action of exogenous Gal-3 delivered into the lungs *via* plasmid versus that of endogenously expressed Gal-3 in the lung. While glycans serve as the predominant ligands for galectins on the cell surface, there is growing evidence that intracellular galectins interact with non-glycan partners to exert their effects (31, 73). This may be yet another reason for the divergent effect noted with Gal-3 delivered into the lungs *via* plasmid. Currently, there are no therapeutic agents/inhibitors targeting endogenous Gal-3 that are commercially available for treatment. Development of selective inhibitors is complicated by the weak nature of the protein–carbohydrate interactions and the extensive sequence homology in the CRD of galectins (74). However, selective Gal-3 antagonists/inhibitors that are effective in attenuating lung fibrosis have been developed that are currently in preclinical or phase I testing (http://glycomimetics.com/galectin-inhibitors/; http://galecto.com/products/galectin-3-inhibitors/).

### Galectin-1

Gal-1 was first identified in electric eels (75). This lectin is a “prototypic” galectin containing a single CRD that can occur as a monomer or a non-covalent homodimer and is found in virtually all adult tissue including lung, liver, brain, kidney, spleen and striated muscle (76). It is expressed by polymorphonuclear cells, macrophages, dendritic cells, activated T cells, stromal cells, endothelial cells, epithelial cells (76, 77) as well as eosinophils (30). Gal-1 is present both inside (nucleus, cytoplasm, and inner surface of plasma membrane) and outside cells (outer surface of cell membrane and extracellular matrix) and as such has intracellular as well as extracellular functions that play a profound role in resolving acute and chronic inflammation by affecting processes such as immune cell adhesion, migration, activation, signaling, proliferation, differentiation, and apoptosis [as reviewed in Ref. (36, 76)]. Increasing evidence from multiple chronic inflammatory disease models such as arthritis, hepatitis, encephalomyelitis, colitis, and nephritis supports the critical anti-inflammatory role of exogenous or endogenous Gal-1 in limiting or resolving inflammation (77). The resolving effects of Gal-1 have also been reported in models of acute inflammation where neutrophil adhesion and transmigration across the inflamed endothelium (78), as well as neutrophil extravasation and mast cell degranulation at sites of inflammation (79) was suppressed. Until recently, little was known regarding the potential role of Gal-1 in allergic asthma. By far the most investigated and well-established role of Gal-1 that may be relevant to allergic inflammation and asthma is the maintenance of T cell homeostasis by virtue of its ability to induce apoptosis of activated T cells and thus control or regulate a strong ongoing immune response [as reviewed in Ref. (36, 80)]. Other known Gal-1 effects that could potentially have a beneficial effect during chronic asthma include induction of IL-10 production by T cells (81, 82) [required for regulatory T cell (Treg)-mediated inhibition of airway inflammation in asthma], supporting inhibitory function of Tregs (83), and suppression of inflammatory cytokine [TNFα and interferon γ] release by T cells (84).

**Figure 3:**
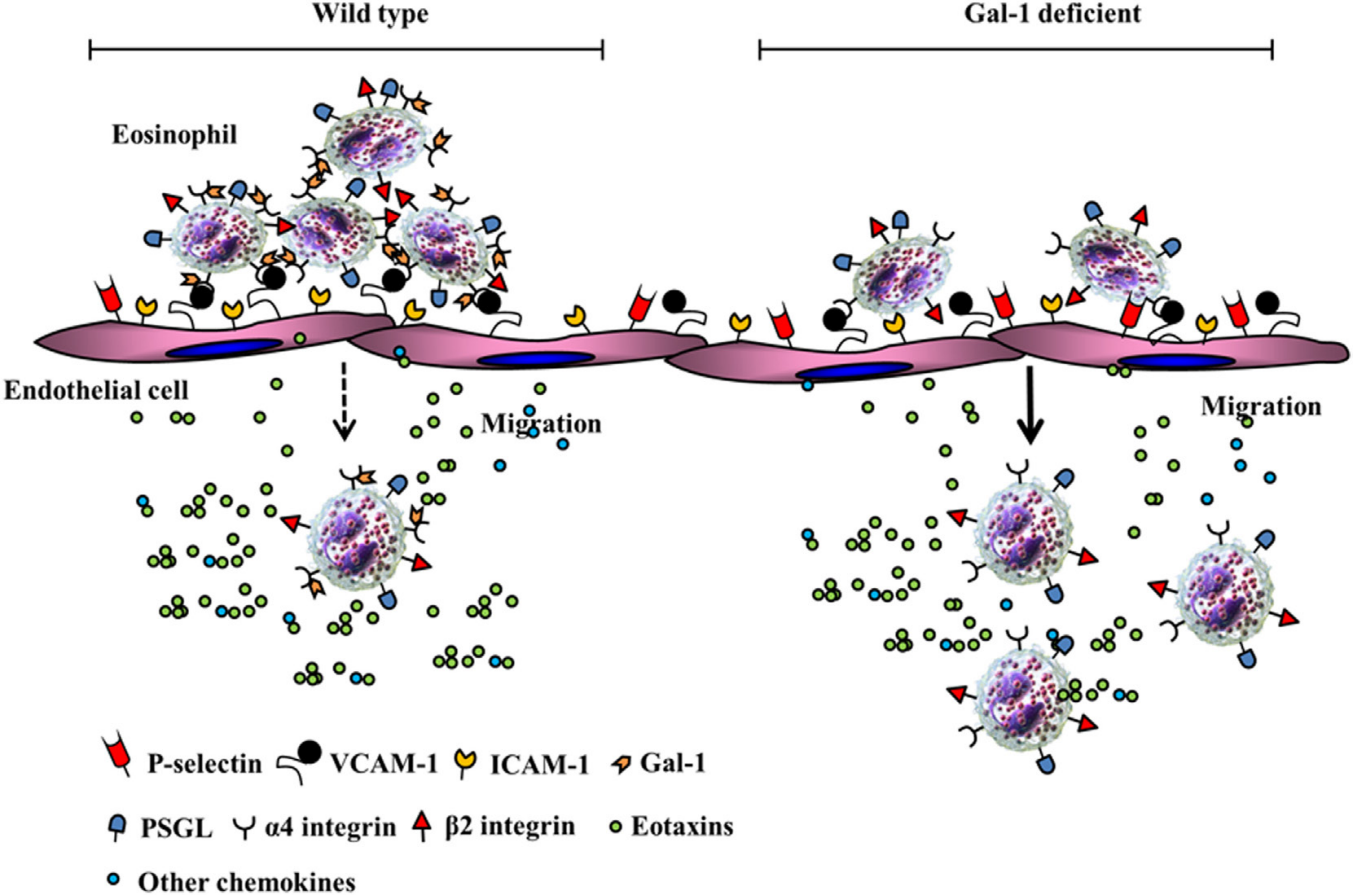
Potential role of Gal-1-α4 interactions in limiting eosinophil trafficking and recruitment. In a wild-type setting, Gal-1 interacts with α4, causes cell clustering, increased adhesion in inflamed blood vessels resulting in restricted chemokine-induced migration. In the absence of Gal-1, eosinophils exhibit decreased adhesion in inflamed blood vessels and increased chemokine-induced migration to extravascular sites of inflammation.

Recent studies from our laboratory demonstrate that allergen-challenged mice deficient in Gal-1 develop more severe airway inflammation (significantly higher eosinophil and T cell infiltration, TNFα level in the lung, and an increased propensity to develop airway hyperresponsiveness) relative to wild-type mice (30). At a cellular level, Gal-1 was found to exert divergent effects on murine bone marrow-derived eosinophils that were *N*-glycan-mediated. At lower concentrations (≤0.25 µM), Gal-1 promoted eosinophil adhesion to VCAM-1 and caused redistribution of α4 integrin to the cell periphery associated with cell clustering/aggregation but inhibited their ability to migrate toward eotaxin-1. While our studies demonstrate that Gal-1 (and Gal-3, described earlier in this review (27)) bind/interact with the α4 subunit of integrin α4β1, they do not rule out the possibility that these galectins can also interact with the β1 subunit. Indeed, previous studies have shown that Gal-1 and Gal-3 can bind to the β1 subunit in other integrin receptors (31, 85). Binding of these galectins to α4 on eosinophils may influence the activation state (resting to active) or conformation of the receptor [as shown in the case of Gal-1 binding to αIIbβ3 in platelets (86)] and enhance cell–cell interaction through receptor clustering/redistribution or bridging through self-association (interaction of α4-bound Gal-1/3 with endothelial-expressed Gal-1/3) (27). Gal-1 binding to integrin receptors (α5β1) has also been shown to affect downstream signaling events, albeit in tumor cells lines (87). Although exposure of eosinophils to Gal-1 leads to reduced activation of ERK1/2 (30), it is unclear at this time whether this effect is dependent on Gal-1-α4 binding. Consistent with the ability of Gal-1 to cause eosinophil aggregation and inhibit eosinophil migration, our studies showed that allergen-challenged wild-type mice had significantly more eosinophils adherent on the endothelium of the blood vessels in lungs but fewer eosinophils in the lung tissue in contrast to allergen-challenged Gal-1-deficient mice that exhibited fewer eosinophils adherent on the endothelium and more eosinophils in the lung tissue. The highlights of our findings pertaining to the role of Gal-1 in eosinophil trafficking are depicted by the schematic shown in Figure 3. Independent of the inhibitory effect of extracellular Gal-1 on cell migration, Gal-1-deficient eosinophils (derived from the bone marrow of Gal-1-deficient mice) showed inherently increased ability to recruit to sites of inflammation *in vivo* relative to wild-type cells, suggestive of a role for intracellular Gal-1 as well in regulating migration. At concentrations (≥1 µM), Gal-1 induced apoptosis in eosinophils and disrupted the cellular actin cytoskeleton leading to decreased levels of F-actin. In a previous study with human peripheral blood eosinophils, immobilized Gal-1 (coated on plastic supports) marginally increased cell adhesion but strongly inhibited migration (relative to P-selectin) and altered actin polymerization/depolymerization dynamics resulting in a prevalence of glomerular actin (i.e., decreased polymerization) (88). Remodeling of the actin cytoskeleton and coordinated polymerization/depolymerization of actin in response to extracellular signals are critical for cellular activities such as cell motility and active processes including cell adhesion and migration (89). The inhibitory effects of Gal-1 on eosinophil migration are likely to be caused by the ability of this lectin to target the actin cytoskeleton as noted in studies by us and others (described above). While allergen exposure causes infiltration of the airways with Gal-1-expressing inflammatory cells and increased soluble Gal-1 in extracellular spaces in the lungs of wild-type mice, this lectin appears to play an essential regulatory role during disease progression by limiting eosinophil recruitment to allergic airways and promoting eosinophil apoptosis, thus suppressing airway inflammation (30). This is further supported by the development of more severe allergic airway inflammation in mice in the absence of Gal-1. Studies have shown that cells (predominantly macrophages) in the sputum of asthmatic patients express lower levels of Gal-1 on the surface relative to cells from healthy donors (90) and propose that decreased Gal-1 levels may contribute to the exacerbated asthmatic immune responses.

The use of Gal-1 as a potential therapeutic (immunosuppressive) agent in Th1- and Th17-mediated inflammatory responses has been investigated in various disease models (91–93). While there are no studies using Gal-1 as a therapeutic agent for allergic asthma, it has been examined in other models of allergic inflammation. Administration of recombinant Gal-1 to mice with IgE-mediated allergic conjunctivitis showed divided results; on the one hand, Gal-1 treatment led to resolution of clinical signs of conjunctivitis and reduced Th2 cytokines (IL-4 and IL-13) and chemokines (eotaxin and CCL5) but resulted in eosinophilia in the conjunctiva with increased Gal-1 expression in the epithelium of the bulbar conjunctiva relative to untreated mice (94). In a model of oral-intestinal allergy syndrome, challenge of mice sensitized to peanut extracts along with Gal-1 showed reduced intestinal allergic inflammation (lower levels of serum histamine, peanut extract-specific IgE and IL-4 with decreased mast cell and eosinophil recruitment in oral and intestinal mucosa) compared to mice sensitized to peanut extracts alone by restoring IL-10 expression in the intestine (95). While findings from these studies are optimistic, it is evident that a better understanding of the functions of extracellular/exogenous versus intracellular/endogenous Gal-1 and its binding partners in these milieus is essential to its utility as a therapeutic agent. Although Gal-1 therapy approaches are being explored, there are challenges to effective therapy, which include stability (monomer–dimer equilibrium, oxidized versus reduced forms) and delivery of intact protein to the site of inflammation (96). In this context, a Gal-1 chimeric molecule with enhanced stability has been developed and shown to alleviate T-cell dependent inflammation in a mouse model of contact hypersensitivity (97). Nonetheless, identifying pathways to induce Gal-1 synthesis and/or favor its biological activity (as in the latter study) might enable exploitation of its pro-resolving function to suppress allergic asthma.

### Galectin-9

Gal-9 was first cloned in 1997 (98) and subsequently isolated from mouse embryonic kidney (99). Like Gal-3, this lectin is widely expressed in many tissues including the lung (99, 100) as well as by immune cells [T cells, B cells, and monocytes (101), eosinophils (102), and dendritic cells (103)]. Gal-9, previously known as ecalectin, belongs to the “tandem-repeat” family of galectins, which contain two conserved CRDs connected by a short peptide domain of varying length (31, 98). Depending on the length of the peptide domain linking the CRDs, three isoforms of Gal-9 have been identified (Gal-9L, Gal-9M, and Gal-9S). Studies by Matsumoto and coworkers in the late 1990s demonstrated that human T cell-derived Gal-9 functions as a potent and specific chemoattractant for human eosinophils *in vitro* and for murine eosinophils when administered intraperitoneally (100). Additionally, antigen stimulation of T cells was found to upregulate Gal-9 expression and release by these cells (104). The CRDs of Gal-9 exhibit high affinity for branched complex *N*-glycans with *N*-acetyllactosamine residues and both CRDs interact with the same or similar ligands on the cell surface of eosinophils to mediate chemotactic activity, with the length of the peptide domain linking the CRDs not being a determinant of this activity (105, 106). Gal-9-induced eosinophil chemotaxis was not mediated *via* binding to the IL-5 receptor or the eotaxin receptor CCR3 (107). Gal-9 has also been shown to activate eosinophils by inducing cell aggregation and superoxide production, but not degranulation (107). Interestingly, differential effects were noted with respect to eosinophil survival. While Gal-9 was shown to prolong survival of normal eosinophils in culture at lower concentrations (≤10 nM) (107), proapoptotic activity was observed when cells were cultured with this lectin at a higher concentration (30 nM) under similar conditions (102). Additionally, Gal-9 suppressed apoptosis of eosinophils from eosinophilic patients but enhanced apoptosis of eosinophils from normal volunteers.

Consistent with its *in vitro* role as an eosinophil chemoattractant, elevated expression of endogenous Gal-9 in the lungs correlated with increased eosinophil recruitment/accumulation in animal models of allergic asthma in guinea pigs (108) and mice (109). Additionally, increased Gal-9 expression associated with increased eosinophil accumulation has also been reported in the nasal polyps of patients with nasal congestion and rhinorrhea (110) and in patients with acute eosinophilic pneumonia (111). While these studies indicate that elevated expression of endogenous Gal-9 may contribute to the pathogenesis of allergic asthma, administration of exogenous Gal-9 has been shown to have the opposite effect resulting in attenuation of Th2-mediated asthma. In a mouse model of allergen-induced airway inflammation, administration of exogenous Gal-9 inhibited airway inflammation by binding to CD44 and preventing CD44-hyaluronic acid interaction, an event that is essential for leukocyte adhesion and migration to the lung (112). Exogenously administered Gal-9 was also shown to suppress airway resistance and eosinophil recruitment in a guinea pig model of allergen-induced airway inflammation (113). Additionally, a suppressive effect was noted on passive cutaneous anaphylaxis in mice, suggestive of a stabilizing effect on mast cells. *In vitro* studies showed that Gal-9 specifically bound to IgE preventing IgE–antigen complex formation and mast cell degranulation (113). Gal-9 has also been shown to induce apoptosis of activated eosinophils, but not non-activated eosinophils, suggesting a potential regulatory function by Gal-9 for activated eosinophils at the site of inflammation (111).

In recent years, the immunoregulatory role of Gal-9 has been widely investigated. In a model of *Ascaris suum*-induced eosinophilic pneumonia, Gal-9-deficient mice exhibited higher numbers of eosinophils and Th2 cells relative to wild-type mice. Interestingly, levels of Foxp3^+^ Tregs were lower. Additionally, administration of exogenous Gal-9 to *A. suum*-exposed wild-type mice prevented eosinophilic inflammation of the lung and increased release of endogenous Gal-9, suggesting an immunoregulatory role for Gal-9 in Th2-mediated eosinophilic inflammation (114). In support of this, cells (macrophages) from sputum of asthmatic patients were found to express lower levels of Gal-9 on the surface than cells from healthy donors, which might be responsible for the exacerbated immune response (90). Studies by Wu et al. have shown that induced Tregs (iTreg) express high levels of Gal-9 and that exogenous Gal-9 plays an important role in maintaining the stability and function of iTreg *via* direct interaction with CD44 (115). Most recently, in a study designed to test the efficacy of Gal-9 as an adjuvant to allergen-specific sublingual immunotherapy in a mouse model of chronic asthma, administration of Gal-9 was found to inhibit eosinophilic airway inflammation, airway hyperresponsiveness, and allergen-specific IgE while inducing transforming growth factor β-1 production as well as the number of CD4^+^CD25^+^Foxp3^high^ Tregs in the BALF, thus suggesting that using Gal-9 as an adjuvant to sublingual immunotherapy may be a more effective treatment option (116). Another interesting finding is that exogenously added Gal-9 suppresses Th17 cell development and expands Foxp3^+^ Tregs from naïve CD4 T cells in an IL-2-dependent manner *in vitro* under “Th17-skewing” conditions (117). This is of importance in the context of those forms of asthma where neutrophils contribute to airway inflammation more than eosinophils (118).

## Other Eosinophil-Expressed Galectins

Gal-10, also known as Charcot–Leyden crystal protein, is a mannose binding, prototypic (i.e., contains one CRD) galectin strongly expressed in human, but not mouse, eosinophils (119). This lectin has the ability to form bipyramidal hexagonal crystals and was identified in the sputum of asthmatics as early as 1872 (120). Several studies have shown that it is associated with eosinophilic inflammation seen in diseases of the airways, and more recently the gastrointestinal tract. Sputum specimens from patients with acute asthma and patients with certain respiratory diseases associated with bronchopulmonary infection have been shown to contain elevated levels of Gal-10 (121). Gal-10 was found to be present in nasal lavage fluid from patients with seasonal allergic rhinitis during allergy season but not before allergy season (122). Overexpression of Gal-10 mRNA was noted in peripheral blood of patients with aspirin-induced asthma compared to patients with aspirin-tolerant asthma despite similarity in other parameters associated with severe asthma in these two groups (i.e., age, peripheral eosinophilia, inhaled corticosteroid use, etc.) (123). More recently, studies have shown that Gal-10 is released in the nasal lavage fluid and expressed at high levels in nasal polyp tissue of patients with aspirin-sensitive respiratory disease relative to aspirin-tolerant asthmatics (124). Genetic variation in the promoter region of the Charcot–Leyden crystal/Gal-10 gene was found to be associated with allergic rhinitis suggesting the possibility that Gal-10 gene transcription may be altered in these individual (125). In another study, Gal-10 concentration in the sputum strongly correlated to the number of eosinophils in the sputum and accurately identified sputum eosinophilia in patients with asthma (126). Apart from its association with eosinophilic disorders of the airways, a direct correlation between Gal-10 protein expression, eosinophil recruitment, and extent of tissue damage has been shown in gut biopsies from patients with celiac disease (127). Along these lines, eosinophils from children with eosinophilic esophagitis had higher levels of Gal-10 mRNA compared to eosinophils from healthy controls (128). Gal-14 is the ovine ortholog of Gal-10, which is expressed specifically by eosinophils and released into the lungs after challenge with house dust mite allergen (129). Because it was found to be released by activated eosinophils and was abundantly present in mucus scrapings from the lung and intestinal tract of sheep after challenge with an allergen or a parasite, this galectin is believed to be secreted by eosinophils at epithelial surfaces and play a role in promoting cell adhesion and changing mucus properties during allergies or parasitic infections *in vivo* (130).

Although Gal-10 has long been considered as an eosinophil-specific protein, later studies showed that Gal-10 is constitutively expressed in the cytoplasm of human CD25^+^ Tregs and is necessary for limiting cell proliferation and Treg-cell-mediated suppression of cocultured CD4^+^ T cells (131). However, many unanswered questions remain regarding the mechanism of suppression, intracellular binding partners of Gal-10, participation of mannose residues, etc. In the context of allergic disease, Gal-10 was recently shown to be associated with atopic dermatitis (132). Serum Gal-10 levels were higher in patients with atopic dermatitis relative to healthy controls, positively correlating with disease severity. Further, Gal-10 was overexpressed in circulating CD3^+^ T cells and IL-22-producing CD4^+^ T cells from atopic dermatitis patients as well as in the skin of chronic atopic dermatitis patients. Overall, the functions of Gal-10 are still elusive; however, the consistent finding that it is associated with eosinophilic inflammatory disorders indicates its potential role as a biomarker for eosinophilic inflammation.

## Concluding Remarks

Eosinophils play a critical role in mediating inflammatory processes in asthmatic lungs by virtue of their ability to release pro-inflammatory cytokines, chemokines, and growth factors that promote development of the hallmark features of asthma. The recruitment of primed mature eosinophils from the blood stream to sites of inflammation in the lung is a multistep paradigm involving initial rolling in the lumen of the blood vessels followed by activation-dependent firm adhesion to the vessel wall and chemoattractant-induced transmigration across the vascular endothelium to extravascular sites, a process driven by cell adhesion molecules (integrins and selectins), chemokines (eotaxin and other chemoattractants), and metalloproteases. In recent years, studies have indicated a regulatory role for galectins in eosinophil trafficking, migration, and activation and thus impact the pathogenesis of allergic asthma. As detailed in this review, Gal-3 plays a pro-inflammatory role in allergic asthma by promoting eosinophil trafficking and migration, while Gal-1 exerts an anti-inflammatory effect due to its ability to limit eosinophil migration and induce apoptosis, wherein decreased expression or absence of this galectin results in increased eosinophilia and exacerbated asthmatic immune responses. The role of Gal-9 in asthma is more intricate; on the one hand, this galectin functions as a chemoattractant for eosinophils and activates these cells but also appears to exert a regulatory function by inducing apoptosis of only activated eosinophils. In disease models, absence of Gal-9 results in increased eosinophilia and Th2 cells but low Foxp3^+^ Tregs while administration of Gal-9 causes an attenuated asthmatic response attributable to induction of endogenous Gal-9 and direct interaction of Gal-9 with CD44 (limiting leukocyte adhesion and migration and promoting stability and function of iTregs).

Studies at a cellular level and in animal models clearly indicate a role for Gal-1, -3, and 9 in regulating (positively or negatively) eosinophil recruitment and the pathogenesis of allergic asthma; however, further studies to elucidate expression patterns of these galectins in relation to different phenotypes and endotypes of allergic asthma in humans are necessary to define whether they can serve as disease biomarkers or therapeutic targets for pharmacological modulation. Currently, there are no therapeutic agents that are commercially available for targeting these galectins endogenously for treatment of asthma. However, considerable progress has been made with respect to Gal-3. Gal-3 has been suggested as a reliable biomarker to predict the modulation of airway remodeling in severe asthma patients before they begin omalizumab therapy (133) and selective Gal-3 antagonists/inhibitors that are effective in attenuating lung fibrosis have been developed that are currently in preclinical or phase I testing. The final outcome of an immune response is often determined by the fine balance of pro- and anti-inflammatory signals. Development of novel forms of Gal-1 and Gal-9 that are stable and can be used for immunotherapy or innovative approaches to induce synthesis or enhance biological activity of these anti-inflammatory galectins may pave the way for future clinical strategies in management of allergic asthma.

## Author Contributions

All the authors listed contributed to the review in close collaboration.

## Conflict of Interest Statement

The authors declare that the research was conducted in the absence of any commercial or financial relationships that could be construed as a potential conflict of interest.
